# Pupils' and teachers' experiences with implementing standing desks in secondary schools in Belgium

**DOI:** 10.1016/j.pmedr.2025.103285

**Published:** 2025-10-21

**Authors:** Veerle Van Oeckel, Benedicte Deforche, Marijke Miatton, Louise Poppe, Maïté Verloigne

**Affiliations:** a Department of Public Health and Primary Care, Ghent University, Corneel Heymanslaan 10, 9000 Ghent, Belgium; b Department of Movement and Sport Sciences, Vrije Universiteit Brussel, Pleinlaan 2, 1050 Brussels, Belgium; c Department of Neurology, Ghent University Hospital, Corneel Heymanslaan 10, 9000 Ghent, Belgium; d Department of Head and Skin, Ghent University, Corneel Heymanslaan 10, 9000 Ghent, Belgium

## Abstract

**Objective:**

Sedentary behaviour is associated with adverse health outcomes in adolescents, yet adolescents spend most of their day sedentary, particularly at school. Standing desks have been proposed to reduce sitting time, but implementation challenges remain. This pilot study evaluated the process of implementing standing desks in secondary schools.

**Methods:**

A mixed-methods study was conducted from September to December 2020 in Flanders, Belgium, in three schools, each including one 7th- or 8th-grade class. Ten standing desks were used for 4–5 weeks in each class. Implementation was assessed retrospectively using pupil focus groups, teacher interviews, and pupil questionnaires. Qualitative data were analysed using reflexive thematic analysis. Questionnaire data were analysed using descriptive statistics.

**Results:**

A convenience sample of 58 out of 68 pupils (mean age 13.0 ± 0.7 years, 44.8 % boys) completed the questionnaire. Pupils reported using the standing desks on average 8.9 ± 5.4 h/week, with qualitative data revealing a decline in use over time. Furthermore, experiences of using standing desks among pupils and teachers varied: some reported better concentration among pupils, while others mentioned discomfort, fatigue, and distractions.

**Conclusions:**

Implementing standing desks resulted in mixed experiences among pupils and teachers. Future interventions should encourage gradual increases in standing time and frequent posture changes.

**Trial registration:**

This study has been registered at ClinicalTrials.gov in January 2020 (NCT04327414; released on March 11, 2020).

## Highlights

• Extensive standing desk use may cause perceived discomfort, fatigue and poor focus.
• Gradually increasing standing time and posture changes may improve acceptability.
• More comprehensive interventions are needed for sustained desk use in classrooms.

## 1 Introduction

Sedentary behaviour negatively affects adolescent health (Chaput et al., 2020), yet European adolescents spend over seven hours per day sedentary (Steene-Johannessen et al., 2020). Schools contribute significantly to adolescents' sedentary behaviour, as 63–80 % of the time at school (Grao-Cruces et al., 2020; Arundell et al., 2019; Egan et al., 2019) and 75 % of the time in class (Arundell et al., 2019) is spent sedentary. Replacing sedentary behaviour with standing has the potential to improve adolescents' health by positively affecting cardiometabolic biomarkers associated with the lipid metabolism (Moura et al., 2019). Although more research has been conducted in primary schools than in secondary schools (Guirado et al., 2021; Rollo et al., 2019), standing desks show promise to reduce adolescents' total sedentary time and prolonged bouts of sedentary time (Schwenke and Coenen, 2022; Sudholz et al., 2016; Sudholz et al., 2023). Moreover, standing desks are considered a relatively easy intervention strategy as it requires little teacher time and expertise (Guirado et al., 2021; Rollo et al., 2019). However, their successful implementation depends on the acceptability and feasibility as perceived by pupils and teachers (Guirado et al., 2021; Rollo et al., 2019). Studies have identified several challenges in implementing standing desks in secondary education. Unlike primary school children, secondary school pupils move between classrooms, making it harder to establish habits to use the standing desks (Sudholz et al., 2023). Additionally, pupils have classes with different teachers, all of whom need to be convinced to encourage the use of standing desks during their lessons (Verloigne et al., 2018). Previous research, involving a classroom equipped with height-adjustable desks and stools for seven weeks, where pupils had the possibility to use the desks for one or two lessons per week, found that some adolescents experienced leg or back pain (Sudholz et al., 2016), which could make them less inclined to use the standing desks. Some teachers in this study also reported a loss of concentration or decreased ability to work effectively among pupils (Sudholz et al., 2016). In a study with a similar setup, but extending over 17 weeks and involving 2–5 lessons per week where pupils could use the standing desks, teachers mentioned that pupils showed more disruptive behaviour (Sudholz et al., 2023). Finally, a loss of concentration was also mentioned by some teachers in a study where three standing desks were added to classes for six months, with pupils using them freely or following a rotation schedule (Verloigne et al., 2018). These negative aspects could make teachers less willing to allow standing desks to be used. These findings highlight the need for process evaluations to determine how standing desks can be implemented in an acceptable and feasible way in secondary education (Guirado et al., 2021; Rollo et al., 2019). Frameworks for process evaluations, such as that proposed by Saunders and colleagues (Saunders et al., 2005), provide a useful basis for this purpose. The framework of Saunders and colleagues includes elements such as the dose delivered (i.e., the amount or number of intended units of each intervention or component delivered by implementers), dose received (i.e., the extent to which participants actively engage with, interact with, are receptive to, and/or use materials), participant satisfaction with the programme, and contextual factors (i.e., aspects of the environment that may influence implementation or study outcomes) (Saunders et al., 2005). Guided by this framework, the present study evaluated the implementation of standing desks in secondary school classrooms from the perspective of pupils and teachers. More specifically, the aim of the study was to assess the intervention dose delivered and received (i.e., the extent to which and how standing desks were used), which were assessed through teacher interviews and pupil questionnaires and focus groups, respectively. Secondly, we aimed to evaluate participant satisfaction with the intervention (i.e., how pupils and teachers experienced the use of standing desks) using the same methods. Finally, we aimed to explore contextual factors that may have influenced the implementation using teacher interviews and pupil focus groups.

## 2 Methods

This process evaluation was embedded within a pilot clustered controlled pre-test post-test trial designed to assess both the implementation of standing desks in secondary school classrooms and, exploratively, the effects on adolescents' cognitive outcomes. The current manuscript only focuses on the process evaluation, as restrictions due to the COVID-19 pandemic impeded adherence to the planned evaluation protocol, thereby precluding a valid outcome evaluation. We used the TIDieR checklist to describe our intervention (Hoffmann et al., 2014) and the COREQ checklist to report the qualitative research conducted for the process evaluation (Tong et al., 2007). The checklists are available in appendix A.

### 2.1 Participants

**Fig. 1:**
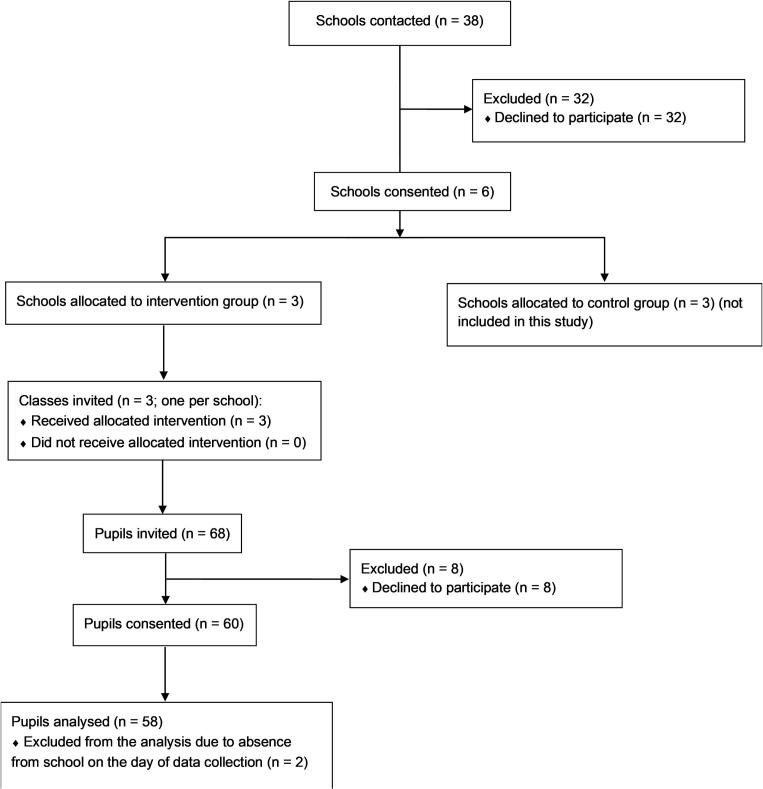
Flowchart of a study examining pupils' and teachers' experiences with implementing standing desks in secondary schools in Belgium in September–December 2020.

A convenience sample of 38 Flemish secondary schools (Flanders is the Dutch-speaking part of Belgium) was contacted between November 2019 and October 2020, via email and subsequently by phone, to participate in this study. The contacted schools offered general education and were located within a 40-min drive distance from Ghent. Six schools (response rate = 15.6 %), of which three intervention schools, agreed to participate in this study. The researchers did not have a connection with any of these schools prior to study commencement. The Flemish education poverty indicator (OKI) for the intervention schools ranged from 0.34 to 1.17 (the average for school year 2020–2021 was 1.08). The OKI is a number between zero and four that indicates the extent to which pupils meet four characteristics (home language non-Dutch, low education level of mother, receiving an education allowance, and living in a neighbourhood with high levels of school delay) with a higher number corresponding to a higher level of poverty. We asked the principal from the intervention schools to select one 7th- or 8th-grade class (mostly 12- to 14-year-olds) from the general education track with at least 20 pupils to participate in the study. The actual number of pupils per class ranged from 19 to 26. As pupils in secondary education in Flanders regularly change classrooms, we also set the requirement that pupils should spend at least half of their classes in the same classroom to ensure the standing desks could be used sufficiently. As a standard secondary school week in Flanders consists of 32 lessons of 50 min each, pupils had to spend at least 16 lessons in the same classroom. All pupils in the selected intervention classes were invited to participate in the study (*n* = 68). A researcher visited the classes to explain the study and hand out the informed consent forms. Informed consent was obtained from 60 pupils and at least one of their parents (response rate = 88 %). A flowchart of the study can be found in Fig. 1. The study was conducted in accordance with the declaration of Helsinki, and received ethical approval from the Ghent University Hospital ethics committee on February 15, 2019 (B670201938818). This study has been registered at ClinicalTrials.gov in January 2020 (NCT04327414; released on March 11, 2020).

### 2.2 Procedure

Between late September and mid-October 2020, ten standing desks were offered to the participating classes for four to five school weeks. The standing desks were from the brand Fully (type ‘Jaswig Nomad’, see appendix B). However, this company was taken over by Herman Miller, and this model is no longer available for purchase. A standing desk is defined as a desk allowing users to work in a standing position without providing support to sit or recline (Guirado et al., 2021). The desks had manual incremental settings to accommodate pupils' height, and included a foot bar. In one school, standing desks replaced traditional desks, while in the two other schools they were added to the classroom setup. In each school, the ten standing desks were placed at the back of a single classroom. Pupils used the desks during lessons held in that classroom, which represented at least half of their classes (an inclusion criterion of this study). We asked schools to set up a rotation schedule to ensure that the standing desks were used as much as possible and that each pupil used the standing desks about equally on a weekly basis. In two schools, this schedule was drafted by the teachers, while in one school this schedule was drafted by the pupils themselves. Consequently, the amount of time each pupil spent using the standing desks varied across schools, depending on the number of pupils per class and the class hours spent in the classroom where the standing desks were located. We also delivered a manual with general information on sedentary behaviour and how to use standing desks in the classroom to the participating schools via email. At the start of the intervention period in one school, teachers observed that pupils struggled to maintain a proper posture at the standing desks, and requested a poster illustrating the correct posture to display in the classroom. In response, researchers designed this poster as an additional supporting intervention component, which was provided to that school at the end of the first intervention week. As the other two schools commenced the intervention one and three weeks later, respectively, the poster was delivered to them during their first intervention week. As all schools received the poster at the start of their intervention period, we consider the potential for confounding due to this additional component to be minimal. After the intervention period (between late October and early December 2020), data for the process evaluation were collected. During this data collection, pupils completed a questionnaire, and one to five weeks later, one or two focus groups with pupils were organised in each school. In addition, teachers' experiences with the standing desks were assessed via interviews (one per school) after the intervention period. Questionnaire data were not processed before conducting the focus groups and interviews.

### 2.3 Data collection

#### 2.3.1 Questionnaire

Questionnaire items provided to the pupils are shown in Table 1. Questionnaire items on the frequency and duration of standing desk use were derived from a previous study on the implementation of standing desks in Belgian primary and secondary schools (Verloigne et al., 2018), with an item added in the present study asking pupils to report standing desk use as the number of lessons per week (each lesson in secondary education in Flanders lasts 50 min). Items assessing determinants of standing desk use (preference, self-efficacy, attitude, habit, and subjective norm in relation to other pupils) were also adapted from Verloigne and colleagues (Verloigne et al., 2018), who based them on the EnRG framework (Kremers et al., 2006; Kremers, 2010). Given the potential influence of teachers, we added an item on pupils' subjective norms related to teachers. The formulations of items on the determinants of standing desk use were based on the ENERGY-child questionnaire and UP4FUN questionnaire, for which psychometric properties were determined in European 10- to 12-year-olds. The respective items in the ENERGY-child questionnaire showed test–retest reliability with ICCs ranging from 0.52 to 0.68 and construct validity with ICCs between 0.19 and 0.45 (Singh et al., 2011). For the UP4FUN questionnaire, the respective items demonstrated test-retest reliability with ICCs ranging from 0.24 to 0.69 (Vik et al., 2015). Finally, we added two multiple-choice items on perceived positive and negative aspects of standing desk use.

**Table 1:** Questionnaire items to conduct a process evaluation among pupils of the implementation of standing desks in secondary schools in Belgium in September–December 2020.

Questionnaire item	Variable	Response options	Recoding
Use of the standing desks in the classroom			
How many times a week do you normally stand at the standing desk (Monday through Friday)?	How often the standing desks were used	1 = never, 2 = almost never, 3 = less than once a week, 4 = once a week, 5 = 2 times/week, 6 = 3 times/week, 7 = 4 times/week, 8 = once every day, 9 = 2 times/day, 10 = 3 times/day, 11 = 4 times/day, 12 = more than 4 times/day	Response options were grouped into four broader categories: never, less than once a week, 1 to 4 times/week, once or more every day.
When you stand at one of those standing desks, how long do you normally stand per turn?	How long the standing desks were used per turn	1 = I didn't use the standing desks, 2 = a half lesson, 3 = a lesson, 4 = more than a lesson	N.A.
How many lessons per week do you normally stand at the standing desk? Give the number of lessons as a number.	How long the standing desks were used, expressed in lessons per week	Open question	N.A.

Determinants of using standing desks in the classroom			
I like to follow lessons while standing at the standing desk.	Preference	1 = I fully disagree, 2 = I disagree, 3 = I'm neutral, 4 = I agree, 5 = I fully agree	N.A.
I find it difficult to follow lessons while standing at the standing desk.	Self-efficacy	1 = I fully disagree, 2 = I disagree, 3 = I'm neutral, 4 = I agree, 5 = I fully agree	This item was recoded so that a higher score on this item represented a higher self-efficacy to use the standing desks (e.g., 1 was recoded to 5).
I think it is good to follow lessons while standing at a standing desk.	Attitude	1 = I fully disagree, 2 = I disagree, 3 = I'm neutral, 4 = I agree, 5 = I fully agree	N.A.
Following lessons while standing at the standing desk is something I do without thinking about it.	Habit	1 = I fully disagree, 2 = I disagree, 3 = I'm neutral, 4 = I agree, 5 = I fully agree	N.A.
Pupils in my class approved it when I followed lessons while standing at the standing desk.	Subjective norm related to other pupils	1 = I fully disagree, 2 = I disagree, 3 = I'm neutral, 4 = I agree, 5 = I fully agree	N.A.
Teachers approved it when I followed lessons while standing at the standing desk.	Subjective norm related to teachers	1 = I fully disagree, 2 = I disagree, 3 = I'm neutral, 4 = I agree, 5 = I fully agree	N.A.
What do you like about using the standing desks? You can mark multiple boxes.	Positive aspects of using standing desks	Multiple-choice question: I can concentrate better, I can better participate in the classroom, it is nice to alternate between following classes while standing and following classes seated, I feel less tired, I have more energy, something else (which could be specified)	N.A.
What do you dislike about using the standing desks? You can mark multiple boxes.	Negative aspects of using standing desks	Multiple-choice question: I can concentrate less, I have pain at my legs/feet, I have pain at my back, standing at the standing desks makes me more tired, it is more difficult to see the blackboard, I feel less involved during classes, something else (which could be specified)	N.A.

Abbreviations: N.A. = not applicable.

#### 2.3.2 Focus groups and interviews

A female researcher (VVO, MSc), trained in conducting qualitative research, performed focus groups with pupils from the participating classes and interviews with teachers giving classes to the participating pupils. A second researcher (master's thesis student), present as an observer, took notes. An overview of the focus groups and interviews is shown in Table 2. In each school, the teacher who served as the researchers' point of contact invited pupils and colleagues to volunteer for the focus groups and interviews. During the focus groups with pupils at schools 1 and 2, a teacher was also present, but did not participate. At the start of the focus group or interview, the interviewer explained its purpose. Next, a semi-structured focus group or interview was conducted using an interview guide containing questions on the following topics: (a) use of the standing desks in the classroom (e.g., “When are the standing desks being used?”), (b) attitude towards using the standing desks (e.g., “In general, how do you feel about having standing desks in the classroom?”), (c) perceived effects of using standing desks (e.g., “How do you feel when you stand at a standing desk? Do you feel certain effects when standing at a standing desk?”), and (d) suggestions for future implementation of standing desks in the classroom (e.g., “Would you like to continue using the standing desks? Why (not)? In what way?”). Detailed topic guides are provided in appendix C. Focus groups and interviews lasted approximately 35–50 min. Focus groups or interviews at school were audio recorded, while online focus groups or interviews were video recorded, after which they were transcribed verbatim.

**Table 2:** Overview of the focus groups and interviews conducted among pupils and teachers evaluating the process of the implementation of standing desks in secondary schools in Belgium in September–December 2020.

School	Pupils or teachers	Number of participants	Online or at school
School 1	Pupils	7	Online
School 1	Teachers	2	At school
School 2	Pupils	7	At school
School 2	Teachers	1	Online
School 3^a^	Pupils	6	At school
School 3^a^	Pupils	8	At school
School 3	Teachers	1	At school

a In this school, two focus groups with pupils were conducted as the focus groups were conducted during class hours, resulting in more pupils that were willing to participate, while in the other schools focus groups with pupils were organised during class-free times.

In addition to the focus groups and interviews, VVO contacted the class teachers of the three participating classes by email and, for schools 1 and 2, a follow-up telephone call, three and a half years after the intervention, to informally ask whether and how the standing desks were still being used. The teachers' responses, provided either by email or during the call, were summarised by the researcher in a Word document.

### 2.4 Analyses

#### 2.4.1 Statistical analyses

Descriptive statistics for the questionnaire data were calculated. Missing data were handled using available case analysis. For continuous variables, descriptive statistics were calculated based on the number of pupils with valid responses. For categorical variables, proportions were calculated relative to the number of pupils who responded to that specific item, rather than to the total sample. Descriptive statistics were calculated using IBM SPSS Statistics (version 29).

#### 2.4.2 Qualitative analyses

To analyse interview and focus group transcripts, a constructivist approach guided the analysis, recognising that reality is subjectively perceived and constructed (Stevens, 2022). As this philosophical paradigm explores people's subjective perceptions and shared meanings people give to a phenomenon (here, the implementation of standing desks in the classroom) (Stevens, 2022), it aligned with our research aim of exploring experiences of pupils and teachers on the implementation of standing desks in the classroom. Following Braun and Clarke's six-phase reflexive thematic analysis (Braun and Clarke, 2006), a single researcher (VVO) conducted the analysis, as themes are understood to be actively generated rather than “discovered”; therefore, inter-rater reliability checks are not expected (Braun and Clarke, 2006). In phase one, VVO familiarised herself with the data by reading the transcripts. In phase two, initial codes were inductively developed at a semantic level by reading through the transcripts and coding relevant sentences or paragraphs. Phase three involved identifying broader patterns of meaning within the codes and forming preliminary themes. During this phase, VVO also made notes on how themes could be related to each other. In phase four, themes were refined to ensure they represented the codes and the overall patterns identified in the data. Phase five involved refining the name of each theme, creating an initial thematic map, and reviewing it with MV and BD before drafting a preliminary report. Finally, in phase six, feedback was sought from all authors to finalise the analysis. During data analysis, the first author (VVO) engaged in ongoing reflection on how her personal background and professional experiences might have influenced the collection and interpretation of the qualitative data, in an effort to minimise bias. For instance, VVO's background in physiotherapy and health promotion, along with her research focus on reducing adolescents' sedentary behaviour and her own use of a sit-to-stand desk at work, may have shaped her expectations regarding the intervention. On the other hand, her close connections with adolescents and teachers in her daily life offered valuable insights that might have enriched the analysis. A more detailed reflexivity statement was added in appendix D. NVivo software (release 14.23.2) was used to analyse interview and focus group transcripts.

## 3 Results

### 3.1 Process evaluation based on questionnaire data

Descriptives of pupils' questionnaire data on the implementation of the intervention are reported in Table 3. Pupils reported to have used the standing desks on average 10.7 ± 6.5 lessons per week. Given that a lesson in Flanders lasts 50 min, this corresponds to 8.9 ± 5.4 h per week. Regarding the determinants of using the standing desks, mean scores on the items ranged from 2.6 ± 1.0 for the habit to use the standing desks to 3.6 ± 1.0 for a positive attitude towards using the standing desks (items were scored on a scale from one to five with a higher score being more favourable to use the standing desks). Finally, the primary aspect pupils liked about using standing desks was that they could alternate between sitting and standing, while more than half of the pupils also reported to have pain in their legs or feet when using standing desks.

**Table 3:** Descriptive statistics of the questionnaire data from pupils on the implementation of standing desks in secondary schools in Belgium in September–December 2020.

Variable	N	Total sample	School 1	School 2	School 3
Number of pupils included in the analysis	–	58	19	24	15
	
Demographics					
Sex (n (%) boys)	58	26 (44.8)	4 (21.1)	15 (62.5)	7 (46.7)
Age in years (mean ± S.D., range)	57	12.97 ± 0.65, 11.73–14.75	13.40 ± 0.35, 12.93–14.29	12.33 ± 0.26, 11.73–12.86	13.50 ± 0.43, 12.86–14.75
	
Questions related to the implementation of the intervention					
How often the standing desks were used (n (%, 95 % C.I.))	58				
Never		1 (1.7, 0–9.2)	1 (5.3, 0.1–26.0)	0 (0, 0–14.2)	0 (0, 0–21.8)
<1 x/week		4 (6.9, 1.9–16.7)	1 (5.3, 0.1–26.0)	0 (0, 0–14.2)	3 (20.0, 4.3–48.1)
1–4 x/week		8 (13.8, 6.1–25.4)	0 (0, 0–17.6)	2 (8.3, 1.0–27.0)	6 (40.0, 16.3–67.7)
≥1 x/day		45 (77.6, 64.7–87.5)	17 (89.5, 66.9–98.7)	22 (91.7, 73.0–99.0)	6 (40.0, 16.3–67.7)
How long the standing desks were used per turn^a^, ^b^ (n (%, 95 % C.I.))	57				
½ lesson		0 (0, 0–6.3)	0 (0, 0–18.5)	0 (0, 0–14.2)	0 (0, 0–21.8)
1 lesson		55 (96.5, 87.9–99.6)	18 (100, 81.5–100)	24 (100, 85.8–100)	13 (86.7, 59.5–98.3)
>1 lesson		2 (3.5, 0.4–12.1)	0 (0, 0–18.5)	0 (0, 0–14.2)	2 (13.3, 1.7, 40.5)
How long the standing desks were used, expressed in lessons per week (mean ± S.D., 95 % C.I.)	58	10.71 ± 6.47, 9.01–12.41	16.11 ± 6.85, 12.81–19.41	9.67 ± 3.55, 8.17–11.16	5.53 ± 4.41, 3.09–7.97
Preference to use the standing desks^c^ (mean ± S.D., 95 % C.I.)	58	3.12 ± 1.09, 2.83–3.41	2.63 ± 0.96, 2.17–3.09	3.46 ± 1.14, 2.98–3.94	3.20 ± 1.01, 2.64–3.76
Self-efficacy to use the standing desks^c^ (mean ± S.D., 95 % C.I.)	58	3.16 ± 1.09, 2.87–3.44	2.84 ± 1.07, 2.33–3.36	3.63 ± 1.10, 3.16–4.09	2.80 ± 0.86, 2.32–3.28
Attitude towards using the standing desks^c^ (mean ± S.D., 95 % C.I.)	58	3.55 ± 1.01, 3.29–3.82	2.95 ± 0.85, 2.54–3.36	3.96 ± 1.00, 3.54–4.38	3.67 ± 0.90, 3.17–4.16
Habit to use the standing desks^c^ (mean ± S.D., 95 % C.I.)	58	2.62 ± 1.04, 2.35–2.89	2.58 ± 1.12, 2.04–3.12	2.96 ± 1.00, 2.54–3.38	2.13 ± 0.83, 1.67–2.60
Subjective norm related to other pupils to use the standing desks^c^ (mean ± S.D., 95 % C.I.)	58	3.22 ± 0.90, 2.99–3.46	2.79 ± 0.85, 2.38–3.20	3.50 ± 1.02, 3.07–3.93	3.33 ± 0.49, 3.06–3.60
Subjective norm related to teachers to use the standing desks^c^ (mean ± S.D., 95 % C.I.)	58	3.00 ± 1.03, 2.73–3.27	2.89 ± 0.99, 2.42–3.37	3.08 ± 1.21, 2.57–3.60	3.00 ± 0.76, 2.58–3.42
Positive aspects of using standing desks (n (%, 95 % C.I.))	58				
It is nice to alternate following classes standing and seated		38 (65.5, 51.9–77.5)	10 (52.6, 28.9–75.6)	18 (75.0, 53.3–90.2)	10 (66.7, 38.4–88.2)
I have more energy		21 (36.2, 24.0–49.9)	7 (36.8, 16.3–61.6)	11 (45.8, 25.6–67.2)	3 (20.0, 4.3–48.1)
I feel less tired		13 (22.4, 12.5–35.3)	5 (26.3, 9.1–51.2)	5 (20.8, 7.1–42.2)	3 (20.0, 4.3–48.1)
I can concentrate better		8 (13.8, 6.1–25.4)	2 (10.5, 1.3–33.1)	2 (8.3, 1.0–27.0)	4 (26.7, 7.8–55.1)
I can better participate in the classroom		7 (12.1, 5.0–23.3)	1 (5.3, 0.1–26.0)	4 (16.7, 4.7–37.4)	2 (13.3, 1.7–40.5)
It is healthier/better for your body^d^		2 (3.4, 0.4–11.9)	0 (0, 0–17.6)	1 (4.2, 0.1–21.1)	1 (6.7, 0.2–31.9)
It is nice to stretch your legs^d^		2 (3.4, 0.4–11.9)	1 (5.3, 0.1–26.0)	1 (4.2, 0.1–21.1)	0 (0, 0–21.8)
I feel fitter^d^		1 (1.7, 0–9.2)	0 (0, 0–17.6)	1 (4.2, 0.1–21.1)	0 (0, 0–21.8)
I have more space^d^		1 (1.7, 0–9.2)	1 (5.3, 0.1–26.0)	0 (0, 0–14.2)	0 (0, 0–21.8)
Nothing^d^		2 (3.4, 0.4–11.9)	1 (5.3, 0.1–26.0)	1 (4.2, 0.1–21.1)	0 (0, 0–21.8)
Negative aspects of using standing desks (n (%))	58				
I can concentrate less		26 (44.8, 31.7–58.5)	7 (36.8, 16.3–61.6)	11 (45.8, 25.6–67.2)	8 (53.3, 26.6–78.7)
I have pain in my legs/feet		34 (58.6, 44.9–71.4)	13 (68.4, 43.4–87.4)	9 (37.5, 18.8–59.4)	12 (80.0, 51.9–95.7)
I have pain in my back		18 (31.0, 19.5–44.5)	6 (31.6, 12.6–56.6)	6 (25.0, 9.8–46.7)	6 (40.0, 16.3–67.7)
Standing at the standing desks makes me more tired		20 (34.5, 22.5–48.1)	8 (42.1, 20.3–66.5)	6 (25.0, 9.8–46.7)	6 (40.0, 16.3–67.7)
I feel less involved during classes		16 (27.6, 16.7–40.9)	5 (26.3, 9.1–51.2)	7 (29.2, 12.6–51.1)	4 (26.7, 7.8–55.1)
It is more difficult to see the blackboard		13 (22.4, 12.5–35.3)	3 (15.8, 3.4–39.6)	4 (16.7, 4.7–37.4)	6 (40.0, 16.3–67.7)
I talk more^d^		1 (1.7, 0–9.2)	0 (0, 0–17.6)	0 (0, 0–14.2)	1 (6.7, 0.2–31.9)
I have a headache from staring at the blackboard^d^		1 (1.7, 0–9.2)	1 (5.3, 0.1–26.0)	0 (0, 0–14.2)	0 (0, 0–21.8)
It was hard to stand up in the first class after cycling to school^d^		1 (1.7, 0–9.2)	0 (0, 0–17.6)	1 (4.2, 0.1–21.1)	0 (0, 0–21.8)
Nothing^d^		1 (1.7, 0–9.2)	1 (5.3, 0.1–26.0)	0 (0, 0–14.2)	0 (0, 0–21.8)

Note: Per-school values not adjusted for class size.

Abbreviations: S.D. = standard deviation, C.I. = confidence interval.

a For this item, we only included responses of pupils who indicated in the previous question to have used the standing desks.

b One lesson in secondary education in Flanders lasts 50 min.

c These items were scored on a scale from one to five with a higher score being more favourable to use the standing desks**.** The only exception was the item on self-efficacy, where a higher score originally indicated lower self-efficacy. This item was recoded so that, in line with the other items, higher scores reflected higher self-efficacy to use the standing desks.

d The questions on what pupils liked and disliked about using the standing desks had an open-ended response option where pupils could add other positive or negative aspects not mentioned in the formulated options.

### 3.2 Process evaluation based on focus group and interview data

**Fig. 2:**
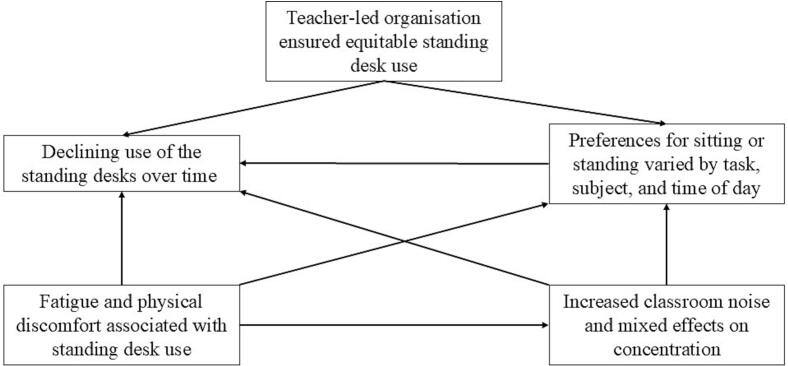
Thematic map showing the experiences of pupils and teachers with implementing standing desks in secondary schools in Belgium in September–December 2020.

This section summarises the findings from the reflexive thematic analysis. The corresponding thematic map is presented in Fig. 2.

#### 3.2.1 Teacher-led organisation ensured equitable standing desk use

When teachers took charge of drafting and implementing the rotation schedule (schools 1 and 2), pupils mentioned the system was consistently applied, and pupils used the standing desks equally. In contrast, when pupils attempted to organise the standing desks use (school 3), they mentioned to struggle to implement their plan (e.g., the rotation schedule was erased from the blackboard resulting in pupils not remembering when to stand up and pupils using the standing desks according to their preferences, or pupils lacked teacher support), leading to inconsistent and unequal standing desks use.

#### 3.2.2 Preferences for sitting or standing varied by task, subject, and time of day

Pupils' and teachers' preferences for sitting or standing varied depending on the task, subject, and time of the day in all schools. Pupils and teachers generally associated sitting with comfort and being less physically demanding, making it the preferred option during stressful moments such as tests, challenging subjects, after physical education, or when pupils felt tired in the morning or evening. In contrast, standing was favoured when it provided a clear advantage, such as during group work, as standing desks were easier to rearrange than traditional desks. However, the pupil focus groups also showed preferences differed between pupils within the participating schools as the perceived effects of sitting or standing differed between pupils (e.g., some found it easier to concentrate while sitting whereas others focused better while standing, or some had more back pain while in others back pain relieved while standing), resulting in some pupils preferring to sit and others to stand during for instance tests. As preferences differed between pupils, pupils from all schools generally expressed a desire for autonomy in deciding when to stand: *“If you're obliged to do that (standing at a standing desk), then it's definitely less fun than if you want to do it yourself.”* (pupil, school 3).

#### 3.2.3 Declining use of the standing desks over time

*“Everyone was super eager to stand up but, as more weeks passed, everyone wanted to sit down a lot more.”* (pupil, school 1), because *“the novelty was kind of gone”* (pupil, school 3). The decline in standing desks use seemed to be primarily reported by pupils from school 3, who used the standing desks according to their preferences in contrast to pupils from schools 1 and 2 who followed a teacher-drafted rotation schedule. Furthermore, the teacher interviews and pupil focus groups showed that the decline was reinforced by contextual factors, such as the placement strategy—standing desks that were added to traditional desks in the classroom (schools 1 and 3) were used less frequently than those replacing traditional desks (school 2). The latter was rather the case in smaller classrooms where space was limited. When standing desks were added to the classroom, pupils mentioned that preferences for sitting or standing and negative experiences, such as discomfort, further contributed to the desks being used less frequently over time.

Moreover, the follow-up phone call three and a half years after the intervention ended, revealed that the standing desks were in a storage room and no longer used in school 1. In school 2, the teacher could not give a conclusive answer on where the standing desks were located, and in school 3, the desks were placed in various locations and used sporadically.

#### 3.2.4 Fatigue and physical discomfort associated with standing desk use

Fatigue and physical discomfort—particularly in the legs and, for some, the back—were the most frequently mentioned physical complaints by the pupils in all three schools. However, experiences varied, as some pupils reported improvements in back pain rather than worsening symptoms. The fatigue and physical discomfort might be explained by two aspects. First, pupils and teachers mentioned it was challenging for pupils to understand what constituted a good posture at the standing desks: *“They were also struggling with how high do I put my desk or how low do I put it?”* (teacher, school 1). Secondly, pupils and teachers across all schools noted the high intensity of use—both in terms of the number of lessons and the duration of standing bouts.

#### 3.2.5 Increased classroom noise and mixed effects on concentration

Especially teachers across the three schools reported increased classroom noise levels: *“The other colleagues actually shared my opinion that it was sometimes more noisy.”* (teacher, school 3). This might be attributed to three main factors. First, teachers reported pupils moved around more when using the standing desks. Second, mainly pupils mentioned that they generally talked more when using standing desks, though they did not always attribute this directly to standing at the standing desks. Some suggested it was *“because you are further away from the teacher”* (pupil, school 3) or because *“you can choose who you sit next to”* (pupil, school 3). Third, the pupils and teachers from schools 2 and 3 mentioned the transitions when pupils rotated in using the standing desks were perceived as too slow by some teachers. This was partly because pupils needed to readjust the desks' height to their own stature (which was especially challenging for smaller pupils), the desks being prone to damage, and COVID-19 measures requiring desks to be sanitised between users. In school 1, this was not an issue because: “*In the afternoon, between the two lessons, there are five minutes. So, before the class started, there was plenty of time to set things right.”* (teacher, school 1).

Secondly, while the interviewed teachers did not observe any effect on pupils' concentration, pupils reported mixed experiences regarding their own concentration when using the standing desks. Some pupils reported improved focus, for example, due to fewer distractions due to increased space between desks. Others, however, found it harder to concentrate. This was again not always attributed to standing at the standing desks itself, but was also caused by increased classroom noise or the positioning of desks at the back of the classroom: *“I personally can't always hear that from the back (of the classroom) and can't always read everything, and then it's difficult to keep up.”* (pupil, school 3). Another pupil said that because pupils are further away from the teacher, *“pupils are more likely to use their phones there”* (pupil, school 3). It was also mentioned that embodied experiences influenced the perceived impact on concentration levels: *“I can concentrate better when I'm standing. Because I'm less tired, I can concentrate better.”* (pupil, school 3).

## 4 Discussion

Unlike previous research, where pupils and teachers were generally positive about standing desks (Guirado et al., 2021), this study revealed rather negative experiences. Nearly 60 % of the pupils reported leg and foot pain, and about 30 % reported more back pain, though pupils in focus groups also reported reduced back pain. Similarly, Sudholz and colleagues found that half of the adolescents (12–16 years) experienced leg or back pain while standing during lessons (Sudholz et al., 2016). Focus groups showed pupils struggled with adopting a correct posture when using standing desks, possibly contributing to musculoskeletal pain. However, it was also mentioned that pupils' posture improved after we provided the teachers with a poster illustrating the correct posture. Future interventions should emphasise a better instruction on a proper posture (Hinckson et al., 2016). Additionally, footwear quality, although not assessed in our study, may affect standing comfort (Orr et al., 2022). Furthermore, the proportion of pupils reporting increased energy levels or feeling less tired was comparable to those feeling more tired when using standing desks. This is contrasting with previous studies that generally found increased energy levels and reduced fatigue among pupils (Sudholz et al., 2016; Verloigne et al., 2018; Kidokoro et al., 2019). Finally, more than three times as many pupils reported decreased concentration compared to those who reported improved concentration. This contrasts with Sudholz and colleagues' findings, where nearly half of the adolescents mentioned improved concentration, while a quarter reported difficulties to pay attention (Sudholz et al., 2016). Pupils in our study attributed concentration issues not necessarily to standing at the standing desks, but also, for example, to their placement at the back of the classroom. This could also explain why about a quarter of the pupils felt less involved during classes when using standing desks. It is therefore important to carefully consider the placement of standing desks in classrooms. In addition, future studies could stratify analyses by desk placement (e.g., front vs. back of the classroom) and account for other contextual factors such as noise levels when examining the impact of standing desks on concentration. Additionally, pupils linked their level of musculoskeletal discomfort and tiredness to their concentration levels. More generally, differences in findings between this and previous studies may plausibly be related to the more extensive use of standing desks in this study (6–16 lessons per week) compared to other research (on average 21.4 min per day (Kidokoro et al., 2019), 60 min per week (Verloigne et al., 2018), or being exposed to sit-to-stand desks for 1–2 lessons per week (Sudholz et al., 2016)). This more extensive use might have caused discomfort, certainly because pupils did not gradually increase their standing time. Prior research suggests standing bouts of 2–5 min may be a feasible starting point for adolescents to gradually increase their standing time (Sudholz et al., 2016). Secondly, the sit-to-stand desks used in some studies allowed more frequent posture changes (Sudholz et al., 2016; Kidokoro et al., 2019), compared to the standing desks used in our study. Frequent posture changes are more beneficial, compared to prolonged static standing, for adolescents' musculoskeletal health (Hinckson et al., 2016) and tiredness (Kidokoro et al., 2019). Future trials that include graded exposure to standing and that vary the “dose” of standing are needed to test these hypotheses. Finally, it is important to note that the data in our study (as well as in the studies we compared with) were reported retrospectively and did not longitudinally examine the effects of standing desks on outcomes such as musculoskeletal discomfort, tiredness, or concentration. To our knowledge, such research has not yet been conducted in secondary education and evidence from primary education is mixed. For example, Ee and colleagues found that Grade 4 boys who used standing desks for 21 school days reported a reduced likelihood of musculoskeletal discomfort in the neck, shoulders, elbows, and lower back compared with those using traditional desks (Ee et al., 2018). Similarly, Parry and colleagues observed among Grade 4 boys using standing desks for a full school year a lower likelihood of discomfort in the neck and shoulders (Parry et al., 2019). In contrast, Koepp and colleagues, who conducted a study among sixth graders using stand-biased desks over eight months (Koepp et al., 2012), and Sherry and colleagues, conducting a study among 9- to 10-year-olds using adjustable sit-stand desks for eight months (Sherry et al., 2020), reported no significant effects on musculoskeletal discomfort (Koepp et al., 2012; Sherry et al., 2020) or concentration (Koepp et al., 2012). Future studies should therefore examine the impact of standing desks on musculoskeletal discomfort, fatigue, and concentration levels in secondary education.

In addition, the process evaluation showed interesting findings to consider when using standing desks in the classroom. As preferences when to sit or stand differed between pupils, they generally favoured to have the autonomy to choose when to stand. A previous study, conducted among 12- to 17-year-olds, showed that younger adolescents responded better to teacher-directed strategies while older adolescents preferred autonomy in choosing when to stand (Sudholz et al., 2023). Secondly, the enthusiasm to use the standing desks waned over time. One of the reasons cited was that the novelty had worn off, which is a trend also noted in previous research (Verloigne et al., 2018; Sherry et al., 2020). Moreover, the follow-up phone call revealed that the standing desks were no longer (regularly) used in any of the schools. This highlights the need for efforts to sustain standing desk use in classrooms in future research. To embed standing desk use in the school culture, changes are not only needed in the structural school environment (the standing desks themselves) but also in the functional school environment (e.g., strategies to ensure teacher support) (Hinckson et al., 2016; Parrish et al., 2018). In addition, while modifying school environments is important, it is equally important to change pupils' motivation to use the standing desks. However, standing desk interventions, including this one, often lack strategies to increase motivation among pupils (Sudholz et al., 2023).

This study was not without limitations. The limited number of schools, due to feasibility constraints, and the use of a convenience sample – comprising the schools, pupils, and teachers participating in the data collection - may limit the extent to which information sufficiency was achieved and the transferability of our findings. Nevertheless, the detailed description of participants and context allows for an informed assessment of the transferability of our findings to other research settings. Secondly, because we did not record the exact number of lessons pupils attended in the classroom with the standing desks, did not objectively verify the adherence to the rotation schedule and/or the time spent using the standing desks (e.g., using accelerometers), we cannot draw definitive conclusions about the fidelity of the intervention's implementation. Furthermore, the short duration of the intervention limits conclusions regarding its long-term sustainability. Another limitation of this study is that the questionnaire was not formally validated. Moreover, focus groups and interviews were conducted both online and in person, which may have introduced mode effects influencing participants' interaction dynamics and responses. In addition, the presence of teachers during some pupil focus groups may have introduced social desirability bias, particularly for behaviours that might be sanctioned, such as phone use or cheating. Furthermore, we did not perform a member check of our transcripts or conclusions based on focus group and interview data. Finally, the COVID-19 pandemic significantly impacted the study. The measures implemented, such as the obligation to sanitise desks and hands when switching positions, likely contributed to negative experiences regarding using the standing desks. A first key strength of this study is that this study was conducted among secondary school pupils, a population that has been less frequently studied in research on standing desks. In addition, we employed a mixed-methods approach, combining questionnaire and interview/focus group data, which allowed us to capture both quantitative and qualitative insights. Moreover, the study included perspectives from both pupils and teachers which broadened our understanding of how standing desks can be implemented in an acceptable and feasible way in secondary education. Finally, the study contributes to open science practices through the sharing of our data on the Open Science Framework.

## 5 Conclusions

Although previous research suggested standing desks in classrooms are a relatively easy intervention strategy to reduce sedentary behaviour among adolescents, this study also reported negative aspects of using standing desks such as musculoskeletal complaints, tiredness, and a worse concentration among pupils. To increase the acceptability of using standing desks in the classroom, future interventions could start with brief standing bouts of 2–5 min and gradually increase duration, embed regular posture changes, provide ergonomics instructions, and carefully consider desk placement (e.g., front or lateral positions). Additionally, blending teacher-directed schedules with student autonomy in using the standing desks may support equitable and sustained use. Lastly, a more comprehensive intervention also targeting the functional school environment (such as teacher support strategies) and pupils' motivation to use standing desks is needed to facilitate a continued use of standing desks in the classroom.

## Availability of data and materials

The dataset generated and analysed during the study is available in the Open Science Framework repository, doi:10.17605/OSF.IO/VG8UK (Van Oeckel, 2025).

## CRediT authorship contribution statement

**Veerle Van Oeckel:** Writing – original draft, Investigation, Formal analysis. **Benedicte Deforche:** Writing – review & editing, Supervision. **Marijke Miatton:** Writing – review & editing. **Louise Poppe:** Writing – review & editing, Supervision. **Maïté Verloigne:** Writing – review & editing, Supervision, Methodology, Funding acquisition, Conceptualization.

## Funding

This work was supported by the 10.13039/501100003130Research Foundation Flanders (FWO) [grant number G072223N]. The funder had no specific role in the study design, collection, analysis and interpretation of data, writing of the report and decision to submit the article for publication.

## Declaration of competing interest

The authors declare that they have no known competing financial interests or personal relationships that could have appeared to influence the work reported in this paper.

## Data Availability

We shared the link to the data in the manuscript.

## Appendix A – D Supplementary data

Supplementary material 1 Appendix A: TIDieR checklist to describe the intervention and the COREQ checklist to report the qualitative research conducted for the process evaluation. Supplementary material 1

Supplementary material 2 Appendix B: Standing desks from the brand Fully (type ‘Jaswig Nomad’) used in this study. Supplementary material 2

Supplementary material 3 Appendix C: Topic guides used for the focus groups and interviews. Supplementary material 3

Supplementary material 4 Appendix D: Reflexivity statement. Supplementary material 4
