# Supplementary material for: Pupils' and teachers' experiences with implementing standing desks in secondary schools in Belgium
Source: Prev Med Rep. 2025 Oct 21;60:103285. doi: 10.1016/j.pmedr.2025.103285 (PMC12594943; doi:10.1016/j.pmedr.2025.103285)
Supplement: Supplementary material 1 — Appendix A: TIDieR checklist to describe the intervention and the COREQ checklist to report the qualitative research conducted for the process evaluation. [file mmc1.docx]

**Appendix A.1.** TIDieR (Template for Intervention Description and Replication) checklist.

| **Item number** | **Item** | **Where located **** | |
| --- | --- | --- | --- |
|  |  | Primary paper  ((sub)title where  the information is  mentioned) | Other ^†^ (details) |
|  | **BRIEF NAME** |  |  |
| **1.** | Provide the name or a phrase that describes the intervention. | ‘Procedure’ |  |
|  | **WHY** |  |  |
| **2.** | Describe any rationale, theory, or goal of the elements essential to the intervention. | ‘Introduction’ |  |
|  | **WHAT** |  |  |
| **3.** | Materials: Describe any physical or informational materials used in the intervention, including those provided to participants or used in intervention delivery or in training of intervention providers. Provide information on where the materials can be accessed (e.g., online appendix, URL). | ‘Procedure’ | The manual and poster are available upon request. |
| **4.** | Procedures: Describe each of the procedures, activities, and/or processes used in the intervention, including any enabling or support activities. | ‘Procedure’ |  |
|  | **WHO PROVIDED** |  |  |
| **5.** | For each category of intervention provider (e.g., psychologist, nursing assistant), describe their expertise, background and any specific training given. | ‘Procedure’ |  |
|  | **HOW** |  |  |
| **6.** | Describe the modes of delivery (e.g., face-to-face or by some other mechanism, such as internet or telephone) of the intervention and whether it was provided individually or in a group. | ‘Procedure’ |  |
|  | **WHERE** |  |  |
| **7.** | Describe the type(s) of location(s) where the intervention occurred, including any necessary infrastructure or relevant features. | ‘Procedure’ |  |
|  | **WHEN and HOW MUCH** |  |  |
| **8.** | Describe the number of times the intervention was delivered and over what period of time including the number of sessions, their schedule, and their duration, intensity or dose. | ‘Participants’, ‘Procedure’ |  |
|  | **TAILORING** |  |  |
| **9.** | If the intervention was planned to be personalised, titrated or adapted, then describe what, why, when, and how. | N.A. |  |
|  | **MODIFICATIONS** |  |  |
| **10.^ǂ^** | If the intervention was modified during the course of the study, describe the changes (what, why, when, and how). | N.A. |  |
|  | **HOW WELL** |  |  |
| **11.** | Planned: If intervention adherence or fidelity was assessed, describe how and by whom, and if any strategies were used to maintain or improve fidelity, describe them. | ‘Data collection’ |  |
| **12.^ǂ^** | Actual: If intervention adherence or fidelity was assessed, describe the extent to which the intervention was delivered as planned. | ‘Process evaluation based on questionnaire data’, ‘Process evaluation based on focus group and interview data’ |  |

** **Authors** - use N/A if an item is not applicable for the intervention being described. **Reviewers** – use ‘?’ if information about the element is not reported/not sufficiently reported.

† If the information is not provided in the primary paper, give details of where this information is available. This may include locations such as a published protocol or other published papers (provide citation details) or a website (provide the URL).

ǂ If completing the TIDieR checklist for a protocol, these items are not relevant to the protocol and cannot be described until the study is complete.

* We strongly recommend using this checklist in conjunction with the TIDieR guide (see *BMJ* 2014;348:g1687) which contains an explanation and elaboration for each item.

* The focus of TIDieR is on reporting details of the intervention elements (and where relevant, comparison elements) of a study. Other elements and methodological features of studies are covered by other reporting statements and checklists and have not been duplicated as part of the TIDieR checklist. When a **randomised trial** is being reported, the TIDieR checklist should be used in conjunction with the CONSORT statement (see [www.consort-statement.org](http://www.consort-statement.org)) as an extension of **Item 5 of the CONSORT 2010 Statement.** When a **clinical trial** **protocol** is being reported, the TIDieR checklist should be used in conjunction with the SPIRIT statement as an extension of **Item 11 of the SPIRIT 2013 Statement** (see [www.spirit-statement.org](http://www.spirit-statement.org)). For alternate study designs, TIDieR can be used in conjunction with the appropriate checklist for that study design (see [www.equator-network.org](http://www.equator-network.org)).

**Appendix A.2.** Consolidated criteria for reporting qualitative studies (COREQ): 32-item checklist.

| **No. item** | **Guide questions/description** | **Reported under title…** |
| --- | --- | --- |
| **Domain 1: Research team and reﬂexivity** | | |
| *Personal characteristics* | | |
| 1. Interviewer/facilitator | Which author(s) conducted the interview or focus group? | ‘Data collection’ > ‘Focus groups and interviews’ |
| 2. Credentials | What were the researcher’s credentials? E.g., PhD, MD. |  |
| 3. Occupation | What was their occupation at the time of the study? |  |
| 4. Gender | Was the researcher male or female? |  |
| 5. Experience and training | What experience or training did the researcher have? |  |
| *Relationship with participants* | | |
| 6. Relationship established | Was a relationship established prior to study commencement? | ‘Participants’ |
| 7. Participant knowledge of the interviewer | What did the participants know about the researcher? E.g., personal goals, reasons for doing the research. | ‘Data collection’ > ‘Focus groups and interviews’ |
| 8. Interviewer characteristics | What characteristics were reported about the interviewer/facilitator? E.g., bias, assumptions, reasons and interests in the research topic. | ‘Data collection’ > ‘Focus groups and interviews’ |

| **Domain 2: study design** | | |
| --- | --- | --- |
| *Theoretical framework* | | |
| 9. Methodological orientation and theory | What methodological orientation was stated to underpin the study? E.g., grounded theory, discourse analysis, ethnography, phenomenology, content analysis. | ‘Analyses’ |
| *Participant selection* | | |
| 10. Sampling | How were participants selected? E.g., purposive, convenience, consecutive, snowball. | ‘Data collection’ > ‘Focus groups and interviews’ |
| 11. Method of approach | How were participants approached? E.g., face-to-face, telephone, mail, email. |  |
| 12. Sample size | How many participants were in the study? |  |
| 13. Non-participation | How many people refused to participate or dropped out? Reasons? | N.A. |
| *Setting* | | |
| 14. Setting of data collection | Where was the data collected? E.g., home, clinic, workplace. | ‘Data collection’ > ‘Focus groups and interviews’ |
| 15. Presence of non-participants | Was anyone else present besides the participants and researchers? |  |
| 16. Description of sample | What are the important characteristics of the sample? E.g., demographic data, date. | N.A. |
| *Data collection* | | |
| 17. Interview guide | Were questions, prompts, guides provided by the authors? Was it pilot tested? | ‘Data collection’ > ‘Focus groups and interviews’ |
| 18. Repeat interviews | Were repeat interviews carried out? If yes, how many? |  |
| 19. Audio/visual recording | Did the research use audio or visual recording to collect the data? |  |
| 20. Field notes | Were ﬁeld notes made during and/or after the interview or focus group? |  |
| 21. Duration | What was the duration of the inter views or focus group? |  |
| 22. Data saturation | Was data saturation discussed? | ‘Discussion’ |
| 23. Transcripts returned | Were transcripts returned to participants for comment and/or correction? | ‘Discussion’ |
| **Domain 3: analysis and ﬁndings** | | |
| *Data analysis* | | |
| 24. Number of data coders | How many data coders coded the data? | ‘Analyses’ |
| 25. Description of the coding tree | Did authors provide a description of the coding tree? | ‘Process evaluation based on focus group and interview data’ |
| 26. Derivation of themes | Were themes identiﬁed in advance or derived from the data? | ‘Analyses’ |
| 27. Software | What software, if applicable, was used to manage the data? | ‘Analyses’ |
| 28. Participant checking | Did participants provide feedback on the ﬁndings? | ‘Discussion’ |
| *Reporting* | | |
| 29. Quotations presented | Were participant quotations presented to illustrate the themes/ﬁndings? Was each quotation identiﬁed? E.g., participant number. | ‘Process evaluation based on focus group and interview data’ |
| 30. Data and ﬁndings consistent | Was there consistency between the data presented and the ﬁndings? |  |
| 31. Clarity of major themes | Were major themes clearly presented in the ﬁndings? |  |
| 32. Clarity of minor themes | Is there a description of diverse cases or discussion of minor themes? |  |
