# Supplementary material for: Pupils' and teachers' experiences with implementing standing desks in secondary schools in Belgium
Source: Prev Med Rep. 2025 Oct 21;60:103285. doi: 10.1016/j.pmedr.2025.103285 (PMC12594943; doi:10.1016/j.pmedr.2025.103285)
Supplement: Supplementary material 2 — Appendix B: Standing desks from the brand Fully (type ‘Jaswig Nomad’) used in this study. [file mmc2.docx]

**Appendix B.** Figures of the standing desks used in the study.

| **Figure B.1.** Jaswig Nomad standing desk used in this study. | **Figure B.2.** Standing desks in one of the classrooms. |
| --- | --- |
| 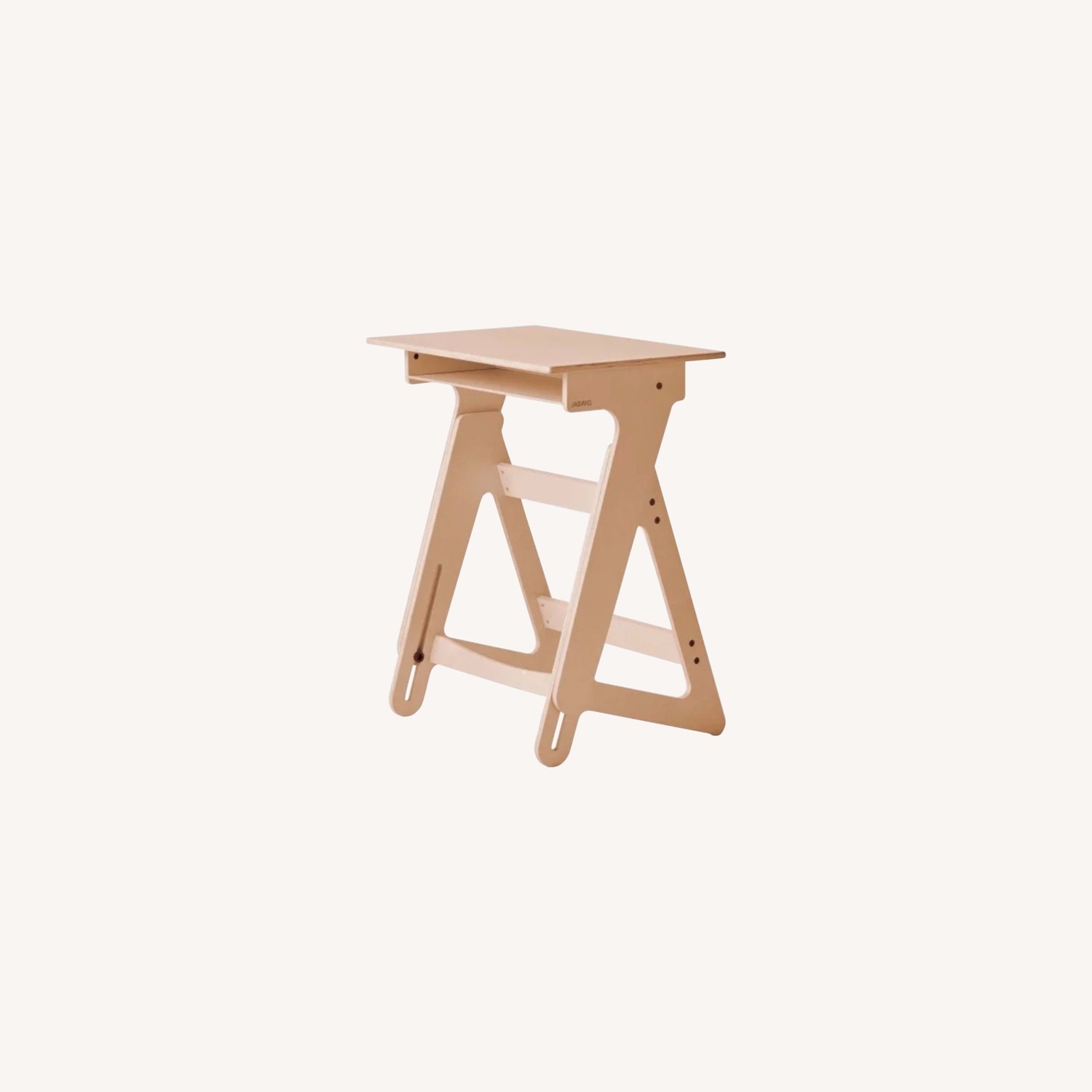 | 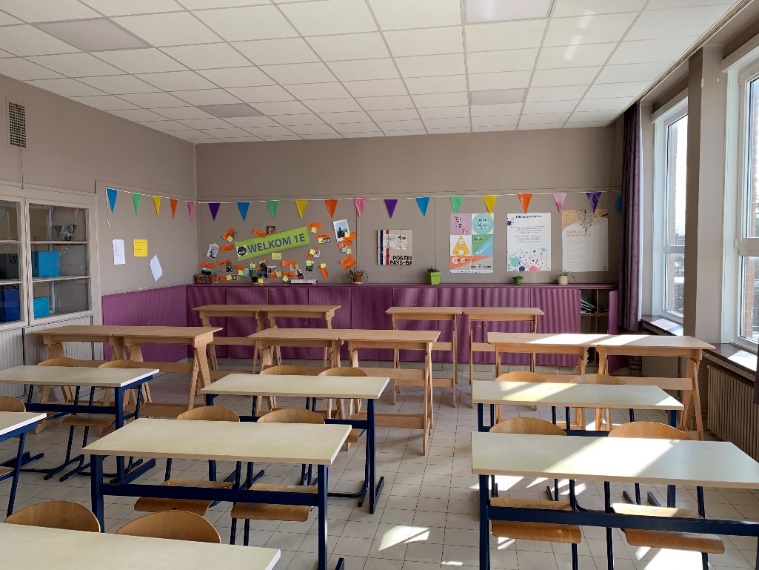 |
