# Supplementary material for: Pupils' and teachers' experiences with implementing standing desks in secondary schools in Belgium
Source: Prev Med Rep. 2025 Oct 21;60:103285. doi: 10.1016/j.pmedr.2025.103285 (PMC12594943; doi:10.1016/j.pmedr.2025.103285)
Supplement: Supplementary material 3 — Appendix C: Topic guides used for the focus groups and interviews. [file mmc3.docx]

**Appendix C.1.** Topic guide for focus groups with pupils to evaluate the process of the implementation of the intervention.

**Questions on the use of the standing desks**

You have 10 standing desks in the classroom.

- How are the standing desks being used in your classroom?
  - When are the standing desks being used?
  - How is it determined who uses the standing desks when?
    - How do you switch positions?
- How long are the standing desks used per turn?
  - - Would you like to spend more or less time at the standing desks? Why?
    - Does everyone spend the same amount of time at the standing desks? Why (not)?
- Do you like the way things were organised? Why (not)?
- Would you like to continue using the standing desks? Why (not)? In what way?
  - Do you think every class or classroom should have standing desks? Why (not)?
  - If you could choose, would you put a few standing desks in the classroom or would you give everyone a standing desks? Why?
  - How would you place the standing desks in the classroom?
  - How much would you use the standing desks?
- Do teachers motivate the use of standing desks? If so, how?
- The standing desks have been there for several weeks now. Is there as much standing as in the beginning, or more or less? Why?
- Are there certain courses or certain tasks for which the standing desks are used more often? Why?

**Questions on the effect of standing desks**

- How do you feel when you stand at a standing desk? Do you feel certain effects when standing at a standing desk?
  - What effect does this have on your concentration?
  - What effect does this have on your fatigue?
  - What effect does this have on your energy levels?
  - What effect does this have on your physical complaints?
- Do you feel that teachers are teaching in a different way because there are standing desks in the classroom? If so, how?
- How do you think the standing desks project affected your sitting behaviour outside the classroom?
- Did standing up more often make you sit down more often at other times or not? Which moments are you talking about? Why do you think that happens?

**Questions about attitudes towards standing desks (advantages, obstacles,...)**

- In general, how do you feel about having standing desks in the classroom?
- What do you like about using the standing desks?/Are there any other things you like?
  - Why do you like that?
- What do you find less good or not good about using the standing desks?/Are there any other things you find less good or not good?
  - Why do you think this is less good or not good?
  - Could you think of a solution for this yourself?
- You have now told some things that are good and not so good about standing desks. If you get to choose now, will you stand at a standing desk or would you rather sit at a regular desk? Why?
- Would you like to have a standing desk at home?
  - Would you use that standing desk? For what? How often?

**Concluding questions**

- Are there any things you would like to share about the standing desks that have not yet been covered in today's conversation?
- Do you have any questions?

**Appendix C.2.** Topic guide for interviews with teachers to evaluate the process of the implementation of the intervention.

**Questions on the use of the standing desks**

There are 10 standing desks in the classroom.

- How are the standing desks being used?
  - When are the standing desks being used?
  - How is it determined who uses the standing desks when?
    - How do pupils rotate? Have you provided a rotation schedule or have you left this to the pupils? Which seems best to you? Why?
  - How long are the standing desks used per turn?
    - Does everyone spend the same amount of time at the standing desks? Why (not)?
- You have now just told us a bit more about how the standing desks are being used. How was this during the last few weeks?
  - What things, if any, have you changed in the way you work over the past weeks?
- Do pupils need to be motivated to stand up/to stand at the standing desks? If so, how do you notice this? If so, how do you do that?
- Do you feel that pupils like standing at the standing desks? From what do you deduce this?
- The standing desks have been there for several weeks now. Is there as much standing as in the beginning, or more or less? Why?

**Questions on the effect of standing desks**

- Do you notice certain effects among the pupils due to the use of the standing desks? If so, which ones and can you explain them a bit more? (What effect do the standing desks have on pupils' concentration/fatigue/energy levels?)
- Have pupils expressed complaints when being taught at standing desks? If yes, which complaints?

**Questions about attitudes towards standing desks (advantages, obstacles,...)**

- In general, how do you feel about having standing desks in the classroom?
- What do you like about using the standing desks?/Are there any other things you like?
  - Why do you like that?
- What do you find less good or not good about using the standing desks?/Are there any other things you find less good or not good?
  - Why do you think this is less good or not good?
  - Could you think of a solution for this yourself?

**Questions on the future implementation of standing desks**

- Would you like to continue using the standing desks? Why (not)? In what way?
  - Do you think every class or classroom should have standing desks? Why (not)?
  - If you could choose, would you put a few standing desks in the classroom or would you give a standing desk to everyone? Why?
  - How many lessons a day at a standing desk do you think is feasible (for pupils)? Why?
- Would you recommend standing desks to other teachers?
- If other teachers were to have standing desks in their classrooms, what tips would you give them?
- In what ways could we support you, teachers, even better?
- What do you think are barriers to implementing standing desks at school? Why?

**Concluding questions**

- Are there any things you would like to share about the standing desks that have not yet been covered in today's conversation?
- Do you have any questions?
