# Supplementary material for: Pupils' and teachers' experiences with implementing standing desks in secondary schools in Belgium
Source: Prev Med Rep. 2025 Oct 21;60:103285. doi: 10.1016/j.pmedr.2025.103285 (PMC12594943; doi:10.1016/j.pmedr.2025.103285)
Supplement: Supplementary material 4 — Appendix D: Reflexivity statement. [file mmc4.docx]

**Appendix D.** Researcher reflexivity.

This reflexivity highlights how VVO's personal background and professional experiences may have shaped the collection and the interpretation of the qualitative data.

The main researcher (VVO) is a female in her late twenties, based in Belgium, with a background in physiotherapy and health promotion. She is a PhD fellow in the health promotion unit at Ghent University, with a genuine interest in school-related sedentary behaviour among adolescents. The aim of her PhD is to investigate strategies to reduce pupils’ sedentary behaviour in secondary schools. At her office, VVO uses a sit-to-stand desk daily, which may have led to expectations regarding the success of such interventions. Being closely connected to adolescents (as a gymnastics coach) and teachers in her personal life gives her some insights into their real-world experiences, though this also required caution to avoid letting prior assumptions influence the interpretation of data.
